# Molecular mechanisms of electron transfer employed by native proteins and biological-inorganic hybrid systems

**DOI:** 10.1016/j.csbj.2020.12.004

**Published:** 2020-12-14

**Authors:** Michael Lienemann

**Affiliations:** VTT Technical Research Centre of Finland Ltd., 02150 Espoo, Finland

**Keywords:** Electron transfer, Electron bifurcation, Redox potential, Redox protein, Enzymatic electrosynthesis, Protein engineering

## Abstract

Recent advances in enzymatic electrosynthesis of desired chemicals in biological-inorganic hybrid systems has generated interest because it can use renewable energy inputs and employs highly specific catalysts that are active at ambient conditions. However, the development of such innovative processes is currently limited by a deficient understanding of the molecular mechanisms involved in electrode-based electron transfer and biocatalysis. Mechanistic studies of non-electrosynthetic electron transferring proteins have provided a fundamental understanding of the processes that take place during enzymatic electrosynthesis. Thus, they may help explain how redox proteins stringently control the reduction potential of the transferred electron and efficiently transfer it to a specific electron acceptor. The redox sites at which electron donor oxidation and electron acceptor reduction take place are typically located in distant regions of the redox protein complex and are electrically connected by an array of closely spaced cofactors. These groups function as electron relay centers and are shielded from the surrounding environment by the electrically insulating apoporotein. In this matrix, electrons travel via electron tunneling, i.e. hopping between neighboring cofactors, over impressive distances of upto several nanometers and, as in the case of the *Shewanella oneidensis* Mtr electron conduit, traverse the bacterial cell wall to extracellular electron acceptors such as solid ferrihydrite. Here, the biochemical strategies of protein-based electron transfer are presented in order to provide a basis for future studies on the basis of which a more comprehensive understanding of the structural biology of enzymatic electrosynthesis may be attained.

## 1 Introduction

**Fig. 1:**
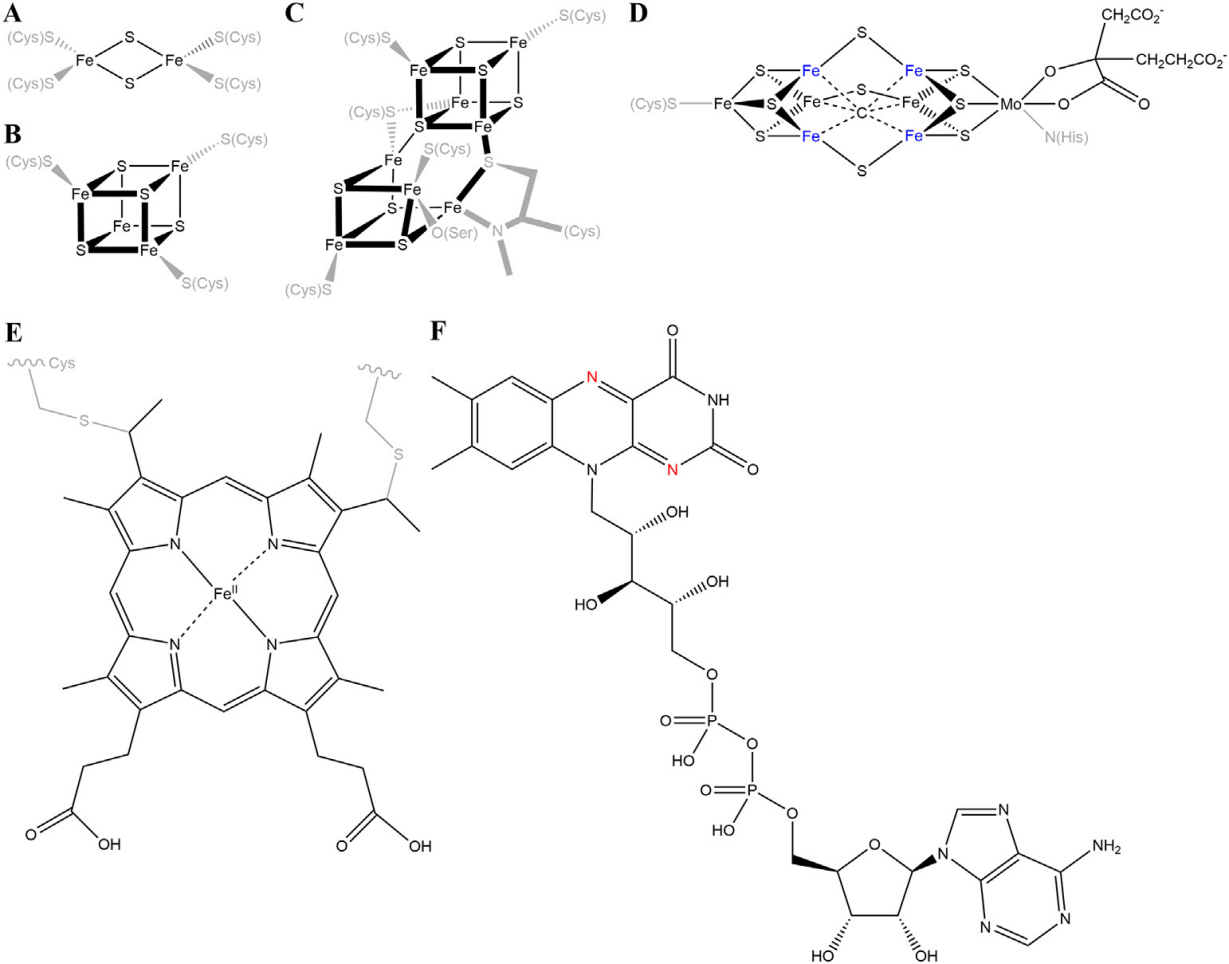
Electron transfer in the apoproteins presented in this review is mediated by various cofactors including the covalently bound iron-sulfur clusters [2Fe-2S] (**A**) and [4Fe-4S] (**B**), the P-cluster (**C**), FeMo cofactor (FeMo-co; **D**), heme *c* (**E**) as well as the non-covalently bound flavin adenine dinucleotide (FAD; **F**). Amino acids contributing to the covalent cofactor attachment to the apoprotein are shown in grey. Catalytically active iron atoms are colored blue and FAD nitrogen atoms transferring electrons during electron bifurcations are displayed in red. (For interpretation of the references to colour in this figure legend, the reader is referred to the web version of this article.)

Survival of each biological species is highly dependent on its ability to harness the energy contained in the chemical bonds in its environment. These reactions are catalyzed by enzymes and power other reactions that ensure the survival and propagation of the cell. Instrumental in this process are electron transfers between intracellular and extracellular compounds, which has been fittingly captured by Nobel Prize-winning physiologist Albert Szent-Györgyi in his famous quote “Life is nothing but an electron looking for a place to rest”. As with most biochemical processes, electron transfer inside proteins is an electron tunneling reaction during which the electron hops from one electron relaying redox center to another, in a stepping stone fashion, until it is transferred to an external electron acceptor (cofactors discussed in this review as redox centers are presented in Fig. 1). A theoretical framework for electron transfer is provided by the Marcus theory with which tunneling rates can be directly predicted for a wide range of reactions [1], [2]. According to this theory, the rate of electron transfer depends on five parameters, i.e., the free reaction energy, Δ*G*^0^, the free activation energy, Δ*G*^‡^, the reorganization energy, *λ*, the electronic coupling of the electron donor–acceptor pair, *H*_DA_, and the temperature. *H*_DA_ is a measure of the probability at which electron tunneling occurs between degenerate states of the donor and acceptor. A large *H*_DA_ is characteristic for an adiabatic electron transfer with a low energy barrier Δ*G*^‡^ when compared to non-adiabatic (low *H*_DA_) electron transfers and strong coupling between the nuclei and electron motion. Δ*G*^0^ is directly determined by the redox potentials of the electron donor–acceptor couple, while *λ* is the energy required to reorganize the nuclei of the surrounding physical medium and increases with donor–acceptor separation. In agreement with the Marcus theory, it was demonstrated by M. Kuss-Petermann et al. that electron transfer reactions are fastest when Δ*G*^0^ and *λ* are of equal magnitude and that reactions are slowed down by an excessively large Δ*G*^0^ [3]. The latter case is referred to as the “inverted regime” and has been subsequently experimentally confirmed using synthetic molecules that perform intramolecular electron transfer [4]. A theory of long-range electron transfer by tunneling in biological systems was developed already in 1974 by J. J. Hopfield [5] and, eight years later, validated experimentally by J. R. Winkler and H. B. Gray. They designed a photochemical system consisting of a photoactive Ru complex as redox center that is covalently bound to the surface of a cytochrome or a blue copper protein [6], [7]. By systematic variation of the position of Ru complex attachment, they were able to estimate *H*_DA_ and *λ*, and determine the pathways along which electrons travel between the redox centers [8], [9]. In the case of electron transfer within proteins, edge-to-edge distances between two individual redox centers do not typically exceed 14 Å thereby ensuring that electron tunneling rates are faster than the common millisecond bond-breaking at the active sites of enzymes [10].

According to a recent survey, iron sulfur clusters and hemes are the most common electron-transferring cofactors in proteins [10], within which these are arranged as precisely spaced arrays that are shielded from the surrounding aqueous environment by an insulating protein matrix [11]. Electron transfer by proteins was initially described for the four membrane complexes of the mitochondrial respiratory chain that transfers two electrons from the reduction equivalents NADH and succinate to a terminal electron acceptor, such as oxygen, using a set of different redox cofactors including flavins, iron sulfur clusters and hemes [12]. The latter two are employed by Complex III and will be discussed in this minireview to illustrate the principles of electron bifurcation.

The possibility of driving enzyme reactions by an electric current at coulombic efficiencies of up to 99% has already been demonstrated for several oxidases and reductases [13] and a vast range of substrates [14]. Here, the catalytic center of the enzyme exchanges electrons with the electrode either directly or indirectly, utilizing stably protein-bound redox cofactors or soluble redox shuttles, respectively. From the perspective of practical application, the direct process appears more appealing since no redox mediator needs to be replenished after removal of the reaction product. However, the stability and efficiency of most tested enzyme systems have been found to be suboptimal for practical applications [13] and would require further development of this technology, possibly with improvement by rational design. This approach requires a comprehensive understanding of the structure–function relationships of electron transferring proteins to which published research has already made many important contributions. This review discusses the concepts and current knowledge of the molecular mechanisms relevant to electron transfer in proteins and highlights some examples of previously described enzyme–electrode hybrid systems.

## 2 Microbial electron transfer systems

### 2.1 Nitrogen reduction to ammonia by the *Azotobacter vinelandii* Mo-nitrogenase

**Fig. 2:**
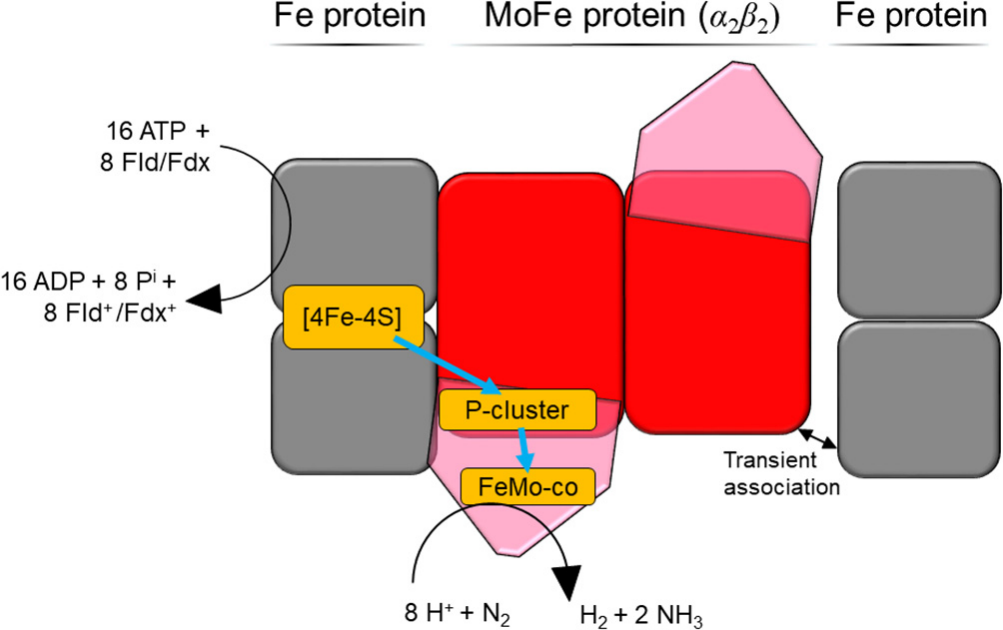
Schematic representation of the *Azotobacter vinelandii* Mo-nitrogenase, displaying the locations of redox cofactors involved electron transfer from soluble electron donors to N_2_.

The reduction of nitrogen is a biologically important process that converts the inert gas into an activated form that is accessible to cell as N-source for the biosynthesis of a wide range of biomolecules such as proteins, DNA and carbohydrates. However, breaking of the triple bond in the nitrogen molecule requires a high amount of chemical energy. In order to perform the highly endergonic nitrogen reduction, nitrogen fixing microorganisms employ the nitrogenase enzyme complex, which consists of a reductase (Fe protein) and an N_2_-reducing subunit (MoFe protein) (Fig. 2). The Fe protein harbors a catalytic cofactor containing a bound iron atom and an additional eponymous metal atom, either Mo, V or Fe. The catalytic mechanism of enzymatic N_2_ reduction has been studied in detail using the Mo-nitrogenase of the model nitrogen-fixing microbe *A. vinelandii* but is still a matter of debate. During the reduction of one molecule of N_2_, at least one equivalent of H_2_ is produced. In the case of productive H_2_ evolution, this reaction supports N_2_ reduction by promoting the binding of the N_2_ molecule and its partial reduction [15]. In addition to this so-called productive H_2_ production, H_2_ can be produced unproductively without concomitant electron transfer to N_2_, as discussed in detail elsewhere [16]. Thereby, the reduction of one N_2_ molecule requires the passage of a minimum of eight electrons through the nitrogenase complex, during which the sequence of events needs to take place eight times (once per electron). The currently debated reaction mechanism is called “Fe protein cycle” and can be described as a ping-pong mechanism. Here, the Fe protein interacts with the β-unit of the MoFe protein through electrostatic interactions and then separates from the MoFe protein to associate with a soluble electron donor (Fld/Flx) using the same surface area. This sequence of events is preceded by a step-wise modulation of the [4Fe-4S]^2+/1+^ cofactor reduction pair potential through ATP binding (*−*0.30 → *−*0.43 V *vs.* SHE) and association of the Fe protein with the MoFe protein (*−*0.43 → *−*0.62 V *vs.* SHE), thus providing the reduction power required to eventually transfer an electron to the catalytic MoCo cofactor [17], [18]. Then, a single electron is transferred to the MoCo cofactor by an initial abstraction from the P-cluster followed by an electron transfer from the [4Fe-4S]^1+^, hydrolysis of 2 ATP, the release of 2 P_i_ and a subsequent electron transfer from ferredoxin or flavodoxin to the Fe-protein cofactor [4Fe-4S]^2+^ [19], [20]. Owing to the soluble electron donor being oxidized as a terminal step, this process is called the “deficit-spending-mechanism” and has inspired engineering efforts, aiming at reducing the P-cluster of the catalytically active protein (i.e., MoFe, VFe or FeFe proteins) by either addition of soluble mediators, covalent attachment of photoelectrocatalytic materials or direct electron transfer from an electrode [21]. These strategies are attractive from an application point of view because they simplify and accelerate the electron transfer reaction by rendering it independent from the Fe-protein-catalyzed ATP hydrolysis and P_i_ release as the rate-limiting step in N_2_ reduction [22].

The role of amino acids in the proximity of the P-cluster and the FeMo cofactor of the MoFe protein of the *A. vinelandii* Mo-nitrogenase has been investigated using mutational analysis [23]. The electroenzymatic reduction of hydrazine (N_2_H_4_) to NH_3_ was quantified using a polyaminocarboxylate-ligated Eu(II) as an electron transfer mediator. The authors found that independent Tyr/His and Phe/His substitutions endowed the enzyme with the ability to electrocatalytically reduce hydrazine while N_2_ reduction was not observed. In 2018, D. P. Hickey *et al.* reported the MoFe-based electroreduction of N_2_ by non-mediated electron transfer to an electrode-bound MoFe protein with concomitant H_2_ reduction [24]. This reaction produced 1.1 µmol NH_3_ per mg of MoFe protein, which highlights the potential of direct electron transfer for the development of enzymatic electrosynthesis applications.

### 2.2 Electron transfer by multiheme cytochromes *c*

Among the known redox proteins, *c*-type multiheme cytochromes (cyts *c*) stand out because, unlike iron-sulfur clusters, they are insensitive to oxygen and are the only known cofactor allowing electron transfer proteins to directly reduce extracellular solid-phase electron acceptors such as Fe(III) and Mn(IV) minerals [25], [26]. The redox cofactor used by multiheme cytochromes is heme *c*, in which a redox-active iron atom is coordinated by a porphyrin molecule in a tetradentate complex (Fig. 1). In cytochromes this iron is present either in the ferrous (Fe^II^) or ferric state (Fe^III^), which have characteristic UV*−*vis absorption maxima that can be monitored spectroscopically in order to follow cytochrome-mediated electron transfer [27], [28]. The porphyrin ring of heme *c* is covalently bound to the apoprotein through two thioether bonds formed with cysteine residues of the characteristic CXXCH heme binding motif of cyts *c* [29].

**Fig. 3:**
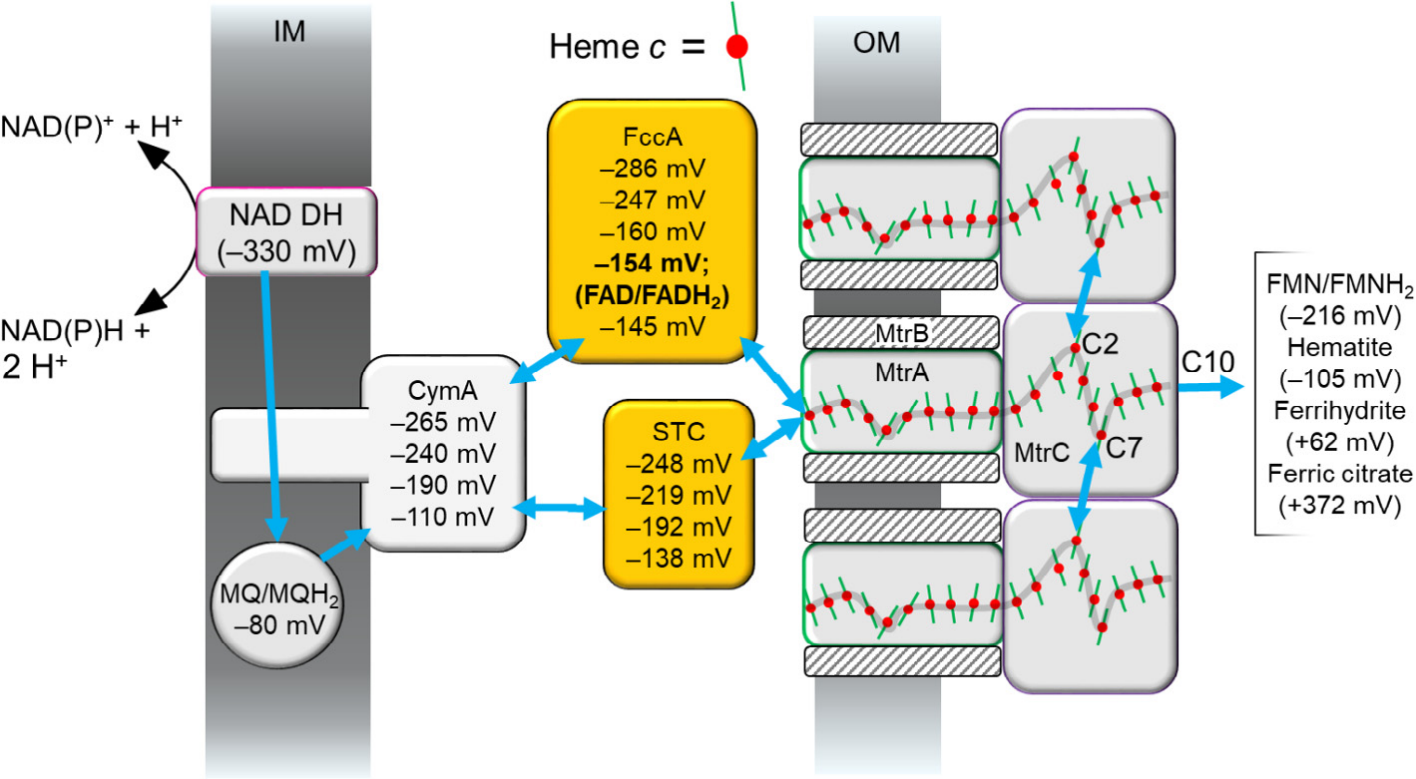
Electron conduit in the cell envelope of *Shewanella oneidensis* connecting the cytosolic face of the inner membrane (IM) and the extracellular face of the outer membrane (OM). Electrons are exchanged between redox cofactors (standard reduction potentials indicated) that are either functional as single-molecule units (menaquinone/menaquinol; MQ/MQH_2_) or bound in soluble cytochromes CymA, FccA and STC (yellow) or membrane-bound electron transfer proteins (grey). Figure modified from [31]. (For interpretation of the references to colour in this figure legend, the reader is referred to the web version of this article.)

Numerous studies have investigated the extracellular electron transfer of the Gram-negative bacterium *Shewanella oneidensis*, establishing it as a model species and providing detailed insights into the molecular mechanisms of this process [26], [30]. According to current understanding, the transmembrane electron transfer proceeds by a multistep incoherent hopping mechanism between redox centers that is initiated at the cytoplasmic face of the inner membrane by extraction of a single electron from NAD(P)H by NAD dehydrogenase (NAD DH). Then, the electron is transferred to a membrane-soluble menaquinone, which in turn reduces the membrane-bound menaquinol dehydrogenase CymA (Fig. 3). CymA passes the electron on either to the periplasmic fumarate reductase (FccA) or to the periplasmic small tetraheme cytochrome (STC), which are soluble proteins and are thereby able to interact with the inner and outer membrane. Upon contact with the inner face of the outer membrane, the reduced periplasmic cytochrome transfers an electron to the transmembrane complex MtrABC. In addition to cytochromes MtrA and MtrC, this complex contains the funnel-shaped porin MtrB which does not actively participate in electron transfer but orients the 185 Å-long heme array of the complex perpendicular to the outer membrane and serves as an insulating sheet to the membrane-spanning MtrA [31]. Both MtrB functions are important for the function of the Mtr complex by, respectively, facilitating optimal electron transfer away from the cell, and shielding the MtrA hemes from membrane-soluble molecules such as oxygen, the reduction of which may give rise to reactive oxygen species causing oxidative damage to the cell. The hemes in the Mtr complex function as stepping stones for the transferred electron, with reduction potentials ranging from 0 to −400 mV. This heterogeneity of the electron transferring cofactors does not, however, restrict the electron transfer in a single direction as it is dependent on the relative redox potentials at the faces of the outer membrane. This was exemplified with purified Mtr complexes in liposome membranes, in which an inward electron transfer occurs when sodium dithionite (*E*^0^′ = *−*660 mV) is present in the exterior and methyl viologen (*E*^0^′ = *−*446 mV) in the interior space [32]. MtrC is bound to the extracellular face of MtrA from which electrons are channeled into the decaheme MtrC by a single heme and transferred to three surface-accessible hemes. This transfer is facilitated by 10 bis-His coordinated hemes that are arranged as a “staggered cross”. The electron transfer to the solid-phase electron acceptor is believed to occur at heme C10 since it is located furthest away from the hydrophobic bilayer core of the OM (~90 Å) and in the vicinity of a putative binding site with a PTPTD amino acid sequence motif [33,28][33], [28]. The remaining surface-accessible hemes C2 and C7 have higher redox potentials than the remaining MtrC hemes and are thus suggested to function as junctions through which electrons are passed on to neighboring cytochromes and facilitate long-range electron transport parallel to the cell membrane [34].

As is valid for all metalloproteins, cytochromes have evolved such that their redox potential corresponds to that of their respective redox partners and can be modulated through alterations of its primary structure. Various factors contribute to the cytochrome redox potential including (in order of decreasing magnitude) the protein matrix, 1^st^ coordination sphere ligands, type of heme deformation and attachment sequence, heme type, heme accessibility, 2^nd^ coordination sphere ligands and surface charges [35]. The magnitude with which these factors modulate the cytochrome redox potential is, however, strongly dependent on the experimental conditions, and is a factor that complicates the estimation of combined effects. Among the mentioned factors, their modulation by stabilization of the heme cofactor in the hydrophobic heme pocket has been investigated using cyts *c*. Here, the low dielectric constant of the protein matrix relative to aqueous solution destabilizes the charged ferric state of the heme iron (Fe^III^) over the neutral ferrous state (Fe^II^) [36]. Cyts *c* span a broad redox potential spectrum that extends to positive redox potentials, which are not reached by cyts with non-covalently bound hemes, such as heme *b*. This is believed to be due to the 10^6^ times weaker binding of the highly charged oxidized heme to the apoprotein when compared to its reduced state, which results in solvation and probably loss of the cofactor [37]. Such dislocation of heme *c* is prevented in cyts *c* by two thioether linkages originating from two Cys residues of the aforementioned CXXCH motif that also directly interacts with the heme Fe^II/III^ atom through the His as proximal ligand. The residues of the highly variable “XX” peptide sequence participate in H-bonding at the axial His, where loss of interactions reduces the histidinate character and increases the redox potential [38]. A counteracting effect of the H-bond network, in terms of the redox potential, is its stabilization of a distorted heme conformation, with respect to a perfectly planar tetrapyrrole ring, that has been demonstrated in case of saddled porphyrins to decrease the reduction potential of the Fe(II)/Fe(III) couple [39], [40]. Hydrophobic and H-bonding interactions involving cyt *c* residues of the 2^nd^ coordination sphere are considered to be crucial determinants of the redox potential and ET reorganization energy, as outlined for the CXXCH motif. Notably, the most prominent structural change observed for cyts *c* upon transition from the ferric to the ferrous state is the reorganization to the H-bond network that extends to the axial Met ligand, as demonstrated for the Tyr67 of yeast *iso*-1-cytochrome *c* [41], [42], [43]. The 1^st^ coordination sphere of the heme iron of natural cyts *c* contains Met at the axial distal ligand position, which influences the redox potential through a combination of endergonic and entropic effects [36]. When considering cytochromes in general, this position can either remain vacant or be occupied by endogenous ligands (e.g., Met or His) or exogenous ones (e.g., H_2_O or OH*^−^*). Most cyts contain endogenous axial ligands, among which a 100*−*150 mV higher redox potential is found for Met ligation when compared to bis-His complexes [44], [45], [46]. The thioether bond of the axial Met ligand has a π-electron-acceptor character with which it stabilizes the ferrous form of the heme iron and is mainly responsible for the high redox potentials of class I cyts *c* [36], [47]. A comparison of microperoxidase-8 and cyt *c* with an unoccupied distal position and a distal Met ligand, respectively, showed that the cyt *c* protein fold efficiently excludes the solvent and thereby renders entropic effects negligible [47].

Multiheme cytochromes are not catalytically active and therefore have not yet been applied for electrosynthetic applications. It is, however, noteworthy that attachment and electrocatalysis has been demonstrated at cathodes and anodes using different heme-containing dehydrogenase and peroxidase enzymes, respectively [14]. Structural studies of cellobiose dehydrogenase revealed that its heme transfers electrons between an external electron donor and the catalytic FAD cofactor and thereby may function as an in-built redox mediator [48], [49]. A comprehensive survey of cytochrome applications involving direct electron transfer is provided in [50].

### 2.3 Flavin-based electron bifurcation in the NADH-dependent ferredoxin:NADP reductase Nfn

Electron bifurcation is a unique case of electron transfer, as it splits the electron influx derived from substrate oxidation to yield one product with a lower and another with a higher reduction potential than the substrate. This is achieved by two half-reactions, which are energetically coupled to drive the endergonic one at the expense of a preceding exergonic transfer [51]. The concept of electron bifurcation was first presented in 1975 to rationalize the electron transfer performed by the quinone-based respiratory chain component complex III (cytochrome *bc_1_*) in the cristal membrane of mitochondria [52]. Essential to understanding the mechanisms of electron bifurcation are the electron transfer reactions yielding and oxidizing the highly energized semiquinone (SQ). Initially, two electrons are transferred from a membrane-soluble hydroquinone (QH_2_) to a complex-III-bound quinone (Q) to yield a bound QH_2_ (*E*^0^′ = +90 mV). The exergonic 1-electron transfer from the bound QH_2_ to soluble cytochrome *c* (*E*^0^′ = +250 mV) via a bound iron sulfur cluster is followed by a movement of the iron sulfur cluster away from the bound SQ that increases the distance between both redox centers to > 20 Å. This structural change favors the second 1-electron transfer “uphill” along the endergonic path from SQ to the low-potential acceptor cytochrome *b*_L_ (*E*^0^′ = −60 mV) [53]. The second electron is then transferred and eventually recycled by transfer to a membrane-soluble Q or QH to yield QH_2_. The spatial separation of SQ and the iron sulfur cluster is a common mechanism among bifurcating flavoproteins by which the high fidelity of electron bifurcation is ensured [54], [55], [56]. In addition, all bifurcating enzymes contain a bifurcating cofactor exhibiting “crossed-over potentials” by which bifurcated electrons are directed into the exergonic and endergonic path at a 1:1 distribution [51]. As opposed to the “normal mode”, where both electrons are transferred to high-potential acceptors, during electron bifurcation, the first transferred electron has a higher potential than the second transferred electron. Here, the first electron transfer produces a highly reactive low-potential SQ radical as a reaction intermediate that is able to reduce a nearby low-potential acceptor.

**Fig. 4:**
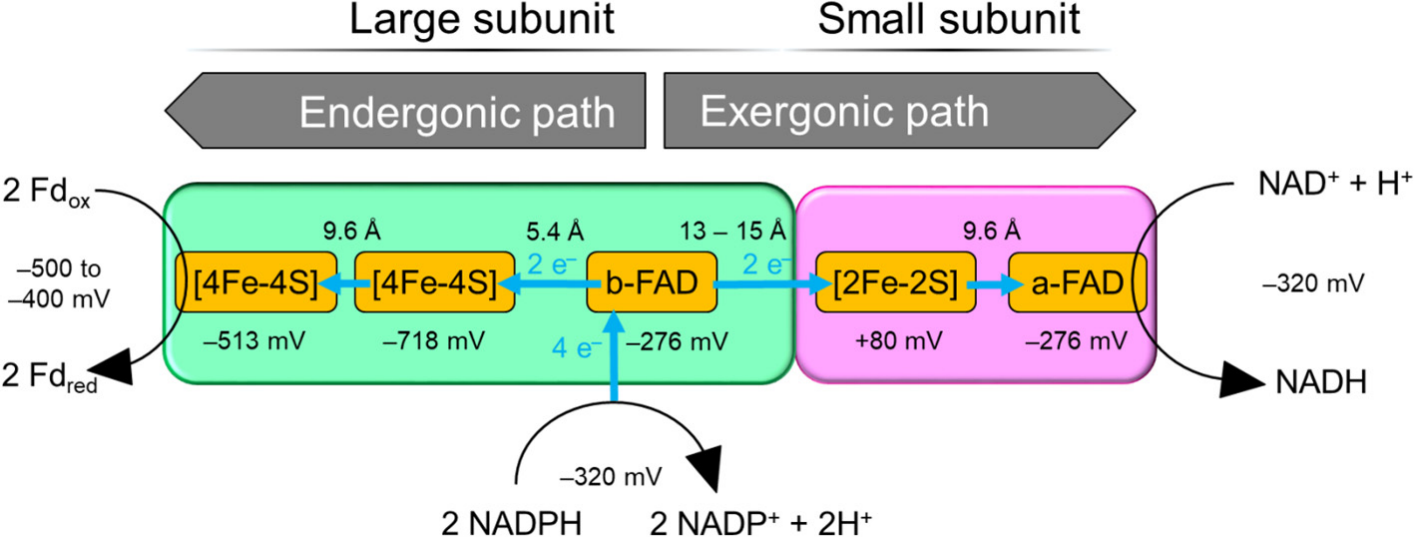
Electron transfer in NADH-dependent ferredoxin:NADP reductase (NfnAB). Edge-to-edge distances between cofactors and mid-point potentials of the enzyme-bound redox cofactors and soluble electron donor and acceptor molecules participating in the electron transfer are indicated as reported in [51].

Until the year 2008, quinones were the only known bifurcating cofactors. This was changed by the discovery of flavin-based electron bifurcation [57], [58]. The bacterial ferredoxin:NADP reductase (NfnAB) is the best-characterized electron bifurcating enzyme complex (Fig. 4) and differs from complex III in that no major conformational changes occur during electron transfer [59], [60]. According to the current understanding of the NfnAB-catalyzed process, electron bifurcation is initiated in the large subunit by a hydride transfer from soluble NADPH to b-FAD. The fully reduced bifurcating cofactor b-FADH is oxidized in a thermodynamically uphill 1-electron transfer to b-FAD^**∙**^^*−*^ (*E*_m,SQ/HQ_ = *−*359 mV) by the high-potential [2Fe-2S]^2+^ cluster (*E*_m_ = +80 mV) [51]. This is followed by an immediate transfer of a second electron from the highly reactive semiquinone FAD^**∙**^^*−*^ (*E*_m,SQ/Q_ = −911 mV) to the proximal [4Fe-4S]^2+^ cluster (*E*_m_ = +80 mV) from which the electron is passed on to the distal [4Fe-4S]^2+^ cluster (*E*_m_ = −513 mV) and transferred further to ferredoxin (*E*_m_ = −500 to −400 mV) as the terminal electron acceptor of the endergonic path. The final steps of the electron bifurcation process occur in the small subunit NfnB, where the [2Fe-2S]^1+^ cluster is oxidized by a-FAD (*E*_m_ = −276 mV) in an endergonic reaction yielding a-FAD^−^ after an additional completion of the aforementioned sequence of electron transfers following the NADPH oxidation. Ultimately, two electrons are transferred from a-FAD^−^ to the soluble electron acceptor NAD^+^ yielding NADH. Thereby, the first electron removal from the reduced flavin b-FADH generates the thermodynamic driving force for the transfer of the second electron into the endergonic path of the NfnAB complex. Furthermore, the five bound redox centers of Nfn illustrate the “14 Å rule” according to which neighboring reduction centers need to be ≤ 14 Å apart to allow for effective electron transfer to the catalytic sites and not limit substrate turnover, which typically takes place in redox enzymes in about one millisecond [10].

**Fig. 5:**
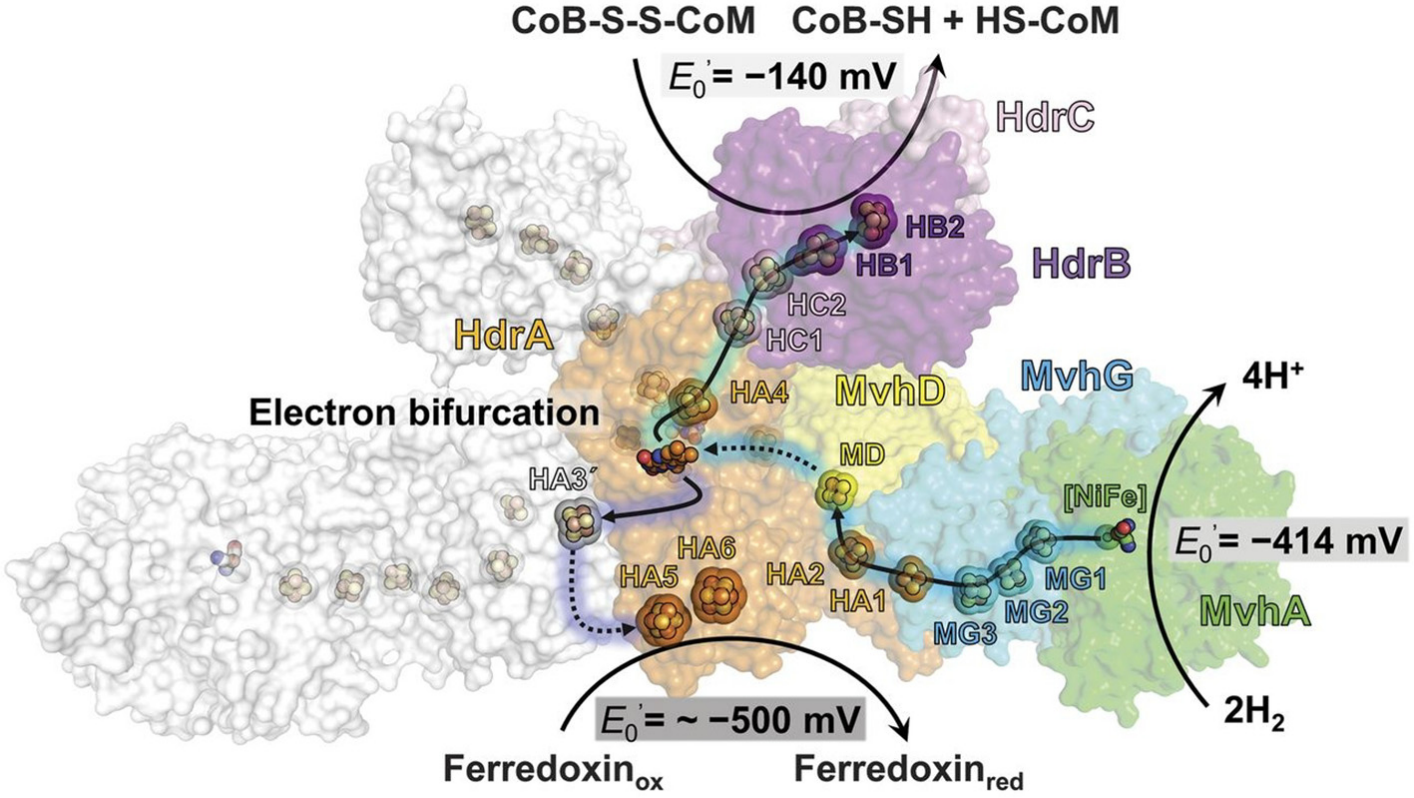
Proposed pathways along which electrons are transferred in *Methanothermococcus thermolithotrophicus* heterodisulfide reductase/hydrogenase MvhADG − HdrABC during oxidation of H_2_ and bifurcated to reduce the CoM-CoB heterodisulfide and ferredoxin. Reproduced from [61] with permission from the American Association for the Advancement of Science.

The recently published structure of the heterodisulfide reductase/hydrogenase MvhADG − HdrABC from the methanogenic archaeon *Methanothermococcus thermolithotrophicus* provided detailed information on the mechanism of electron bifurcation during methanogenesis utilizing an extended array of iron sulfur clusters [61], (Fig. 5). This enzyme oxidizes H_2_ and transfers the extracted high-potential electron to coenzymes M and B and the low-potential electron to ferredoxin using a bifurcating flavin and an array of 14 iron sulfur clusters. According to the available structural data, the two isoalloxazine rings of the flavin are separated > 30 Å from the closest cofactor of the electron transfer route originating at the site of H_2_ oxidation ([2Fe-2S] cluster MD). The apparent MD–FAD distancing violates the 14 Å rule since it exceeds the permissible length for electron tunneling and, based on high B factors, conformational rearrangements have been proposed to occur during electron transfer that reduce the MD–FAD spacing. Another noteworthy heterodisulfide reductase-containing complex is the *Methanococcus maripaludis* heterodisulfide reductase supercomplex, which contains two electron transfer routes spanning the heterodisulfide reductase HdrABC, with the bifurcating FAD bound to HdrA, and an attached hydrogenase domain VhuD as a constant component. The electron transfer routes are extended by either a formate dehydrogenase FdhAB or an auxiliary hydrogenase VhuAG that are incorporated during growth under H_2_ limitation or H_2_ excess, respectively [62], [63]. Enzymatic electrosynthesis of formate at 90% efficiency was demonstrated using the (Fdh)_2_ homocomplex, but the redox cofactors and molecular mechanisms involved in this reaction require further investigation [64].

## 3 Concluding remarks

The discussed electron-transferring proteins allow their host cells to perform vital anabolic and catabolic reactions by accessing distant electron acceptors, conservation of chemical energy or accessing essential nutrients that require chemical activation. As apparent from the electron bifurcating flavoproteins and the wire-like multiheme cytochromes, dynamic changes of the protein structure can support the catalysis of redox reactions but were not essential for electron transfer over long distances. In addition, the transient association of the nitrogenase protein complex components effectively facilitated the gradual supply of chemical energy required for the highly endergonic electron transfer from flavodoxin/ferredoxin to the inert gaseous substrate. Furthermore, the structure-aided engineering of this complex facilitating electrosynthesis of H_2_ and NH_3_ serves as a proof-of-principle for the possibility to employ engineering approaches for the advancement of the enzymatic electrosynthesis technology. The knowledge gained until now on the functionality of the protein structure for electron transfer on a molecular level motivates further studies employing experimental and computational methods for the design of novel biological-inorganic hybrid systems.

## Declaration of Competing Interest

The authors declare that they have no known competing financial interests or personal relationships that could have appeared to influence the work reported in this paper.
